# Scale-adaptive model for detection and grading of age-related macular degeneration from color retinal fundus images

**DOI:** 10.1038/s41598-023-35197-2

**Published:** 2023-06-13

**Authors:** Niveen Nasr El-Den, Ahmed Naglah, Mohamed Elsharkawy, Mohammed Ghazal, Norah Saleh Alghamdi, Harpal Sandhu, Hani Mahdi, Ayman El-Baz

**Affiliations:** 1 Department of Computer and System Engineering, Faculty of Engineering, Ain Shams University, Cairo, Egypt; 2 Department of Bioengineering, University of Louisville, Louisville, KY USA; 3 Electrical, Computer and Biomedical Engineering Department, College of Engineering, Abu Dhabi University, Abu Dhabi, United Arab Emirates; 4 Department of Computer Sciences, College of Computer and Information Sciences, Princess Nourah Bint Abdulrahman University, Riyadh, Saudi Arabia

**Keywords:** Biotechnology, Computational biology and bioinformatics, Biomarkers, Diseases, Engineering, Mathematics and computing

## Abstract

Age-related Macular Degeneration (AMD), a retinal disease that affects the macula, can be caused by aging abnormalities in number of different cells and tissues in the retina, retinal pigment epithelium, and choroid, leading to vision loss. An advanced form of AMD, called exudative or wet AMD, is characterized by the ingrowth of abnormal blood vessels beneath or into the macula itself. The diagnosis is confirmed by either fundus auto-fluorescence imaging or optical coherence tomography (OCT) supplemented by fluorescein angiography or OCT angiography without dye. Fluorescein angiography, the gold standard diagnostic procedure for AMD, involves invasive injections of fluorescent dye to highlight retinal vasculature. Meanwhile, patients can be exposed to life-threatening allergic reactions and other risks. This study proposes a scale-adaptive auto-encoder-based model integrated with a deep learning model that can detect AMD early by automatically analyzing the texture patterns in color fundus imaging and correlating them to the vasculature activity in the retina. Moreover, the proposed model can automatically distinguish between AMD grades assisting in early diagnosis and thus allowing for earlier treatment of the patient’s condition, slowing the disease and minimizing its severity. Our model features two main blocks, the first is an auto-encoder-based network for scale adaption, and the second is a convolutional neural network (CNN) classification network. Based on a conducted set of experiments, the proposed model achieves higher diagnostic accuracy compared to other models with accuracy, sensitivity, and specificity that reach 96.2%, 96.2%, and 99%, respectively.

## Introduction

Age-related macular degeneration (AMD) is a retina disease that affects the retina’s macular region, a part of the retina that controls sharp straight-ahead vision^1^, causing progressive loss of central vision^2^, and may lead to complete visual disability^3^. AMD happens when aging causes damage to the macula. Dry AMD and Wet AMD are the two primary forms of AMD; each has different grading. Dry AMD also called Atrophic AMD or non-neovascular AMD, has three grades: early, intermediate, and late, also called geographic atrophy (GA) or advanced non-neovascular AMD. Wet AMD, also called exudative^4^ or neovascular AMD, is always late stage and has two grades: inactive and active^5^. Moreover, wet AMD can be further classified into classic, occult or mixed^6^. Neovascular and late dry are considered advanced AMD^7^. The hallmark of AMD is the drusen formation that is an accumulation of retinal deposits, pigmentary changes at the macula that serves as a predictor of more advanced AMD development^4,8^ and mild to moderate vision loss^7^. Change in size and number of drusen indicates AMD progression risk^8^ and grading characteristics^9^. Dry AMD is the most common form, although wet AMD is less frequent but is responsible for 90% of blindness due to AMD^7^.

AMD is the cause of 87% of blindness cases worldwide^10,11^, where Europeans recorded the highest prevalence over Asians in early and late AMD over Africans in any AMD. Statistically, in 2014^10^ anticipated that new cases of AMD would reach 196 million in 2020 and by 2040 this number will reach 288 million globally, while^12^ predicted that in 2050, the number of early AMD cases would be 39.05 million and late AMD will be 6.41 million. AMD is a chronic disease and neither of its forms can be cured^13^. However, treatment for wet AMD can help maintain and even improve vision, or halt the disease’s development^14^. Early detection can help prevent disease progression; however, any dry AMD stages can turn into wet AMD. Traditionally the clinical diagnosis of the disease requires examination and assessment of either fundus autofluorescence imaging or optical coherence tomography (OCT) supplemented by fluorescein angiography or OCT angiography without dye^3,6^ or Spectral Domain Optical Coherence Tomography (SD-OCT).

During the past few years, much deep learning (DL) approaches have been applied in computer vision (CV) tasks including medical imaging classification, due to its robust architecture and better performance. DL models record good results in retinal image analysis for detecting and diagnosing retinal diseases like AMD, glaucoma, choroidal neovascularization (CNV), and diabetic macular edema (DME) based on different imaging modalities such as retinal fundus images, OCT, SD-OCT. In the literature, several studies have tried to classify and discriminate between AMD’s different grades and normal retinas. Rivu Chakraborty and Ankita Pramanik proposed a novel deep convolutional neural network (DCNN) architecture with 13-layers to classify non-AMD and AMD based on fundus images^15^. The model is composed of five convolutional layers (CL), five max-pooling layers (MPL), and three fully connected layers (FCL) training on the iChallenge-AMD dataset. The model recorded 89.75% accuracy without data augmentation, while applying 4-time and 16-time data augmentation versions, the model recorded 91.69%, and 99.45% accuracy respectively. They also trained their model on the ARIA dataset and recorded accuracy of 90%, 93.03%, and 99.55% for original, 4-time data augmentation, and 16-time data augmentation respectively. In Ref.^16^ the authors proposed a multiscale CNN with 7 CL for binary classification of AMD and standard images using OCT images. The generated model is trained on the Mendeley dataset and achieved high accuracy between 99.73 and 96.66% when tested on different datasets like Mendeley OCTID, SD-OCT Noor dataset, and Duke. Several authors^17–19^ reported high accuracy and good performance on AMD classification based on OCT images. References^20–23^ are some of the state-of-the-art deep learning architectures for AMD classification where Refs.^20,21^ used transfer learning to apply different classification problems for AMD grades while Tan et al.^22^ used 14-layer DCNN with data augmentation to increase the size of the iChallenge-AMD training dataset to perform binary classification between AMD and normal retina recording accuracy of 89.69%.

Based on OCT imaging datasets^24–29^ applied transfer learning using different pre-trained models to detect and classify AMD. Xu et al.^24^ used the ResNet50^30^ model recording an accuracy 83.2%. Hwang et al.^25^ used different pre-trained models such as VGG16^31^, InceptionV3^32^, and ResNet50 models to identify AMD types into normal, dry AMD, active wet, and inactive wet, it recorded accuracies of 91.40%, 92.67%, and 90.73%, respectively. Yoo et al.^26^ used the VGG-19 pre-trained model and random forest classifier recording an accuracy of 82.6% training the DL model recorded an accuracy of 83.5% when trained on the fundus imaging dataset and 90.5% when combining the usage of the fundus with OCT imaging datasets. Chen et al.^27^ used transfer learning to classify OCT images of AMD and DME and recorded VGG19, Resnet101, and Resnet50 among seven pre-trained models recording average accuracies of 99.42%, 99.19%, and 99.09%, respectively. Wang et al.^28^ applied transfer learning using a VGG19 pre-trained model to classify AMD grades and differentiate between AMD, GA, drusen, and normal images, where the model accuracy of 93.14% using OCT images collected from Northwestern Memorial Hospital of total 498 OCT images. Serener et al.^29^ compared ResNet18 with AlexNet to distinguish between dry and wet AMD OCT images where ResNet18 recorded better performance with an accuracy of 99.5% while AlexNet recorded an accuracy of 81%.

Auto-encoders^33^ are an artificial neural network that attempts to convert inputs into outputs with the least amount of acceptable distortions by compressing input data into a lower-dimensional representation before reconstructing the original data from this compressed representation. It extracts informative features and useful characteristics from data while filtering out noise and irrelevant information^34^. Auto-encoders may be used for data compression^35^ in which the compressed representation is used to keep the information in a more compact format, as well as denoising, in which the model is trained to reconstruct clean data from noisy input. It can also be used for image-to-image translation^36^ by randomly sampling from the compressed representation and decoding it to generate a new image as well as dimensionality reduction^37–40^ by training an auto-encoder, the network can learn a compressed representation of the data that captures the most important features and capable of generating new images such as variational auto-encoder (VAE)^41^. In the auto-encoder-based model, the latent space layer is responsible for performing dimensionality reduction. the encoder performs a dimension reduction operation by translating the input into lower-dimensional representation in accordance with the decoder. In general, auto-encoders are then trained to reconstruct original, noise-free data from the given input data.

We aim to build an automated model that can easily discriminate between normal retinas (no-AMD), intermediate dry AMD, GA, and wet (neovascular) AMD grades with high accuracy based on fundus images while overcoming the challenge of having different fundus image dimensions stimulates building customized image resizing generator based on CNN model that automatically generates resized image to $224 \times 224$ px that could also be integrated with any pre-trained model and correctly discriminate between the previously mentioned four grades. Consequently, our customized model will take care of any needed data preprocessing before training starts. We would like to highlight two major contributions in the proposed CAD system. The first contribution is the development of a new scale-adaptive auto-encoder-based model that can integrate with any pre-trained network while retaining critical information from the original data. It is worth noting that this contribution is not limited to the proposed application but can be utilized for scaling down any input data to match the input of a pre-trained network, enabling transfer learning. The second significant contribution is the creation of a comprehensive CAD that can effectively differentiate between AMD and normal cases, and grade AMD patients into three categories: Intermediate, GA, and Wet. To the best of our knowledge, we are the first research group to provide such a comprehensive assessment of AMD from fundus images. To highlight this contribution, we conducted numerous experiments based on transfer learning using our dataset.

The rest of the paper is organized as follows: “Material and methods” section introduces the proposed model methodology related to the research, explaining the auto-encoder-based scale adapting network and the classification network. The “Experiments and results” section shows the results recorded for the conducted set of experiments. The “Discussion” section explains the obtained results. Finally, the “Conclusion and future work” section presents the conclusion and outlook for future work.

## Material and methods

**Figure 1:**
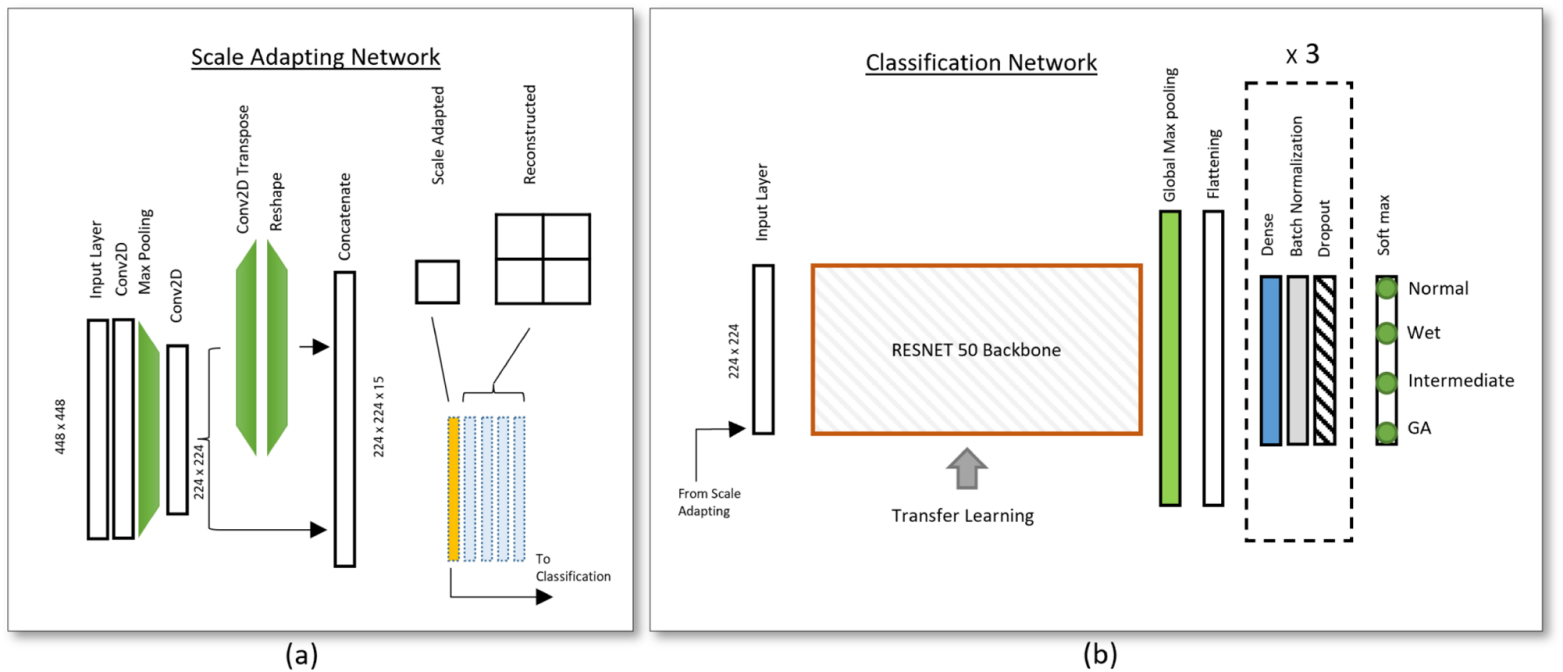
(**a**) Schematic diagram of the Auto-Encoder inspired architecture proposed for the scale adapting network. (**b**) The schematic diagram for the proposed classification network for the detection and grading of AMD. The output layer is Softmax with one-hot encoding nodes corresponding to normal, wet, intermediate, and GA AMD grades.

This study aims to provide a solution for the classification problem distinguishing between AMD grades by classifying colored fundus images of patients that are either normal or have intermediate AMD, GA or wet AMD grades. The method is applied to a local dataset. Our proposed model is an integrated model between two stages. First stage is a custom auto-encoder-based model that takes the fundus images as its input from the available dataset and feeds its output to the second stage which is a ResNet50 pre-trained model. Figure 1 shows the proposed integrated model diagram.

### Data collection

A cohort of 864 human subjects was recruited for this study by The Comparisons of Age-Related Macular Degeneration Treatments Trials (CATT), sponsored by the University of Pennsylvania^42^. This study was available for those aged 50 and older. During the two years of the clinical trial, 43 clinical centers in the United States enrolled participants who received intravitreal injections of ranibizumab or bevacizumab and one of three dosing regimens. All imaging and clinical data for this study were de-identified by the CATT Study Group before being sent to the University of Louisville. Because the data had been collected in the past by a third party and had been appropriately de-identified, it was deemed to be exempt from the local institutional review board (IRB) process by the IRB of the University of Louisville. All data collection methods were carried out in accordance with relevant guidelines and regulations. Informed consent was obtained from all subjects and/or their legal guardian(s). The CATT program provided study treatments on every participant’s first visit. Treatment was delivered to those in the fixed monthly dosing groups every visit or as needed based on the presence of exudation. Treatment evaluations were conducted every visit for those assigned to variable dosing groups. Participants who had lesion activity received study treatment. From these data, we collect 216 normal, 216 intermediate AMD, 216 GA AMD, and 216 Wet AMD.

### Auto-encoder based scale adapting network

Regarding the in-equal fundus images sizes, we built our customized resizing model that accepts any fundus image size (as large as $2224 \times 1888$ px to $547 \times 491$ px) and resizes it to $224 \times 224$ px to be used in applying transfer learning on any pre-trained model. The scale adapting (SA) network is an auto-encoder-based neural network model, accordingly it filters out noise and irrelevant information. The auto-encoder-based model aims to resize the input images to $224 \times 224 \times 3$ dimensions and take care of any needed data preprocessing before classification training starts. It is constructed of two CL and a MPL after which a split of two branches takes place where a branch is made of a CL, transpose convolutional layer (TCL), and finally, a reshape layer that reshapes its output to $224 \times 224 \times 12$. In the end, the two paths are combined using the concatenation layer to produce a work containing high and low-resolution images of $224 \times 224 \times 15$ dimensions. The required output dimension is obtained from the low resolution that is generated from the first branch while the high resolution is needed to ensure that the output is the same as the original input image. During training, the model learns to minimize the reconstruction error between the input and the generated output image by applying the custom loss function comparing low and high-resolution output images with the input image. The high-resolution image is the reconstructed image that is supposed to be as similar to the actual input image, while the low-resolution image is the required resized output.

**Figure 2:**
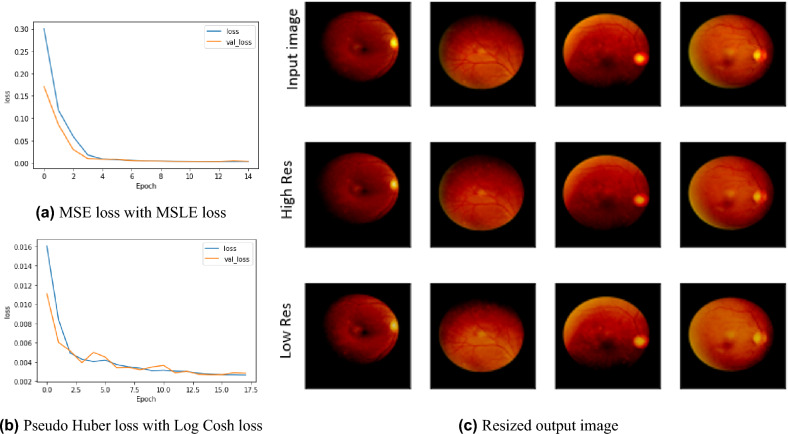
(**a**) and (**b**) shows the loss curve over training epochs for two different loss function and (**c**) shows the output results for high and low resolution and the effects of resized generator custom model.

We used Adam optimizer with a fixed 0.001 learning rate and tanh as the activation function. The training was then performed over 100 epochs with a batch size equal to 1. Our custom scale-adaptive (SA) auto-encoder-based model recorded a perfect match regenerated image of 1 structural similarity index measure (SSIM) using a combination of two loss functions Pseudo Huber loss function and Log Cosh loss function for high resolution and low resolution respectively, proofing good quality recording Root Mean Square Error (RMSE) 0.081. By trying different combinations between Mean Square Error (MSE) loss function and Mean Square Logarithmic Error (MSLE) loss function, our model showed efficiency and recorded 1 SSIM while RMSE enhanced to 0.075. This comparison was fairly evaluated with the same hyper-parameters, using an Adam optimizer with a 0.0001 learning rate, setting the factor of the high-resolution loss function to 0.25. In contrast, the low-resolution loss function factor was set to 0.075. Figure 2 shows the experimental results for our SA model, where Fig. 2a shows the loss function curve over training epochs for using the combination of MSE and MSLE losses for high resolution and low resolution respectively, while Fig. 2b shows the loss function curve over training epochs for using the combination of Pseudo Huber loss function and Log Cosh loss function for high resolution and low resolution respectively. Figure 2c shows the output results for our SA model using the combination of MSE and MSLE losses for high resolutions and low resolutions respectively.

### Classification network

The proposed classification network architecture is shown in Fig. 1b. It is constructed of a ResNet50 convolution backbone, a global average pooling layer, flatten layer, three repeated blocks, and a final softmax dense layer. Each block is architected: a dense layer, a batch normalization layer to stabilize and speed up the training process, and a dropout layer to avoid overfitting. All of the dense layers use the Rectified Linear Unit (ReLU) as its activation function setting all values less than zero to 0, and retaining all the values greater than zero, except for the last dense layer uses softmax as the output layer with four nodes to represent normal (no AMD), intermediate, GA and wet AMD grades. We used categorical cross-entropy as the loss function, stochastic gradient descent (SGD) optimizer starting with a 0.001 learning rate that was reduced automatically during the training phase to improve results whenever the loss metric has stopped improving, a total of 24,750,212 out of 24,811,338 parameters were used for training the proposed classification network architecture.

Due to dataset size limitation, we applied transfer learning, where we used the ResNet50 pre-trained model based on the weights of the ImageNet dataset. The training was performed over 300 epochs with a batch size of 64. The dataset samples were split into 70% for the training set and the remaining 30% for validation and testing sets.

While carrying out training on a limited number of samples, we applied data augmentation on the training dataset to increase its size and avoid overfitting by implementing the following data augmentation process: image rotation by rotating the image at $50^{\circ }$ angle, and image mirroring by flipping the image horizontal and vertical data augmentation is only applied during the training phase and no augmentation used during the testing phase, this leads to train the model among samples and test against the remaining samples.

## Experiments and results

The proposed model was trained on Colab-Pro GPU. We developed, trained, validated, tested our model, and calculated its performance metrics in python using TensorFlow^43^, Keras^44^, and scikit-learn^45^, the later along with matplotlib^46^ and seaborn^47^ were used for plotting all of the shown figures and graphs such as performance metrics, confusion matrix, feature extraction, and activation map. We applied k-fold cross-validation technique to validate the best model performance and propose our model that is composed of our SA model integrated with ResNet50 model. The hyperparameters have been set for each model separately where the scale adaptive auto-encoder-based model hyperparameters were set as follows: batch size is 1, Adam optimizer with a fixed 0.001 learning rate, and tanh as the activation function while the ResNet50 pre-trained model hyperparameters were set as: batch size 64, SGD optimizer with automatic adaptive learning rate starting with 0.001 and reduced whenever the accuracy evaluation metric stops improving.

### Accurate detection and grading compared to other models

**Figure 3:**
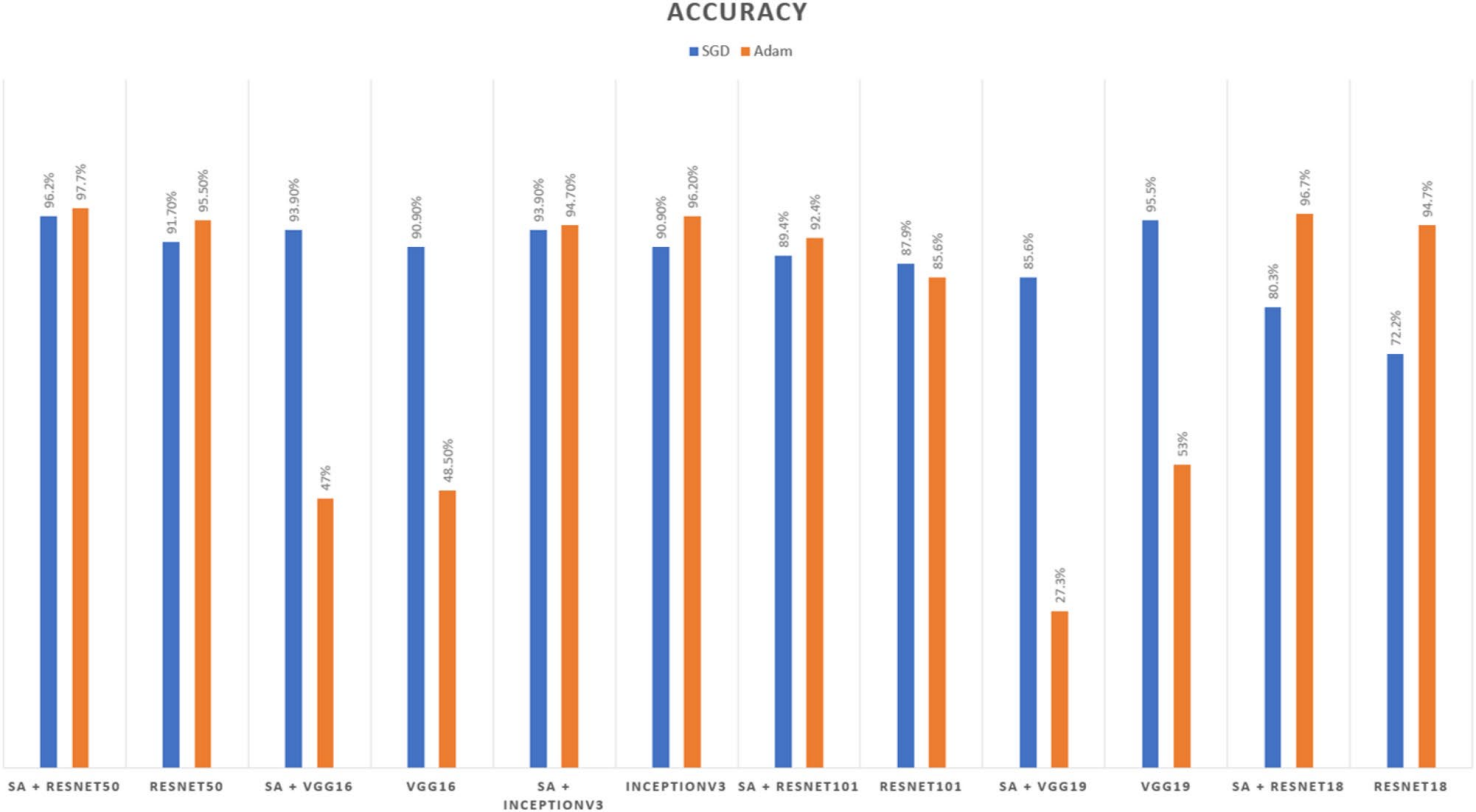
Comparison of models’ accuracy for using SGD optimizer and Adam optimizer.

**Figure 4:**
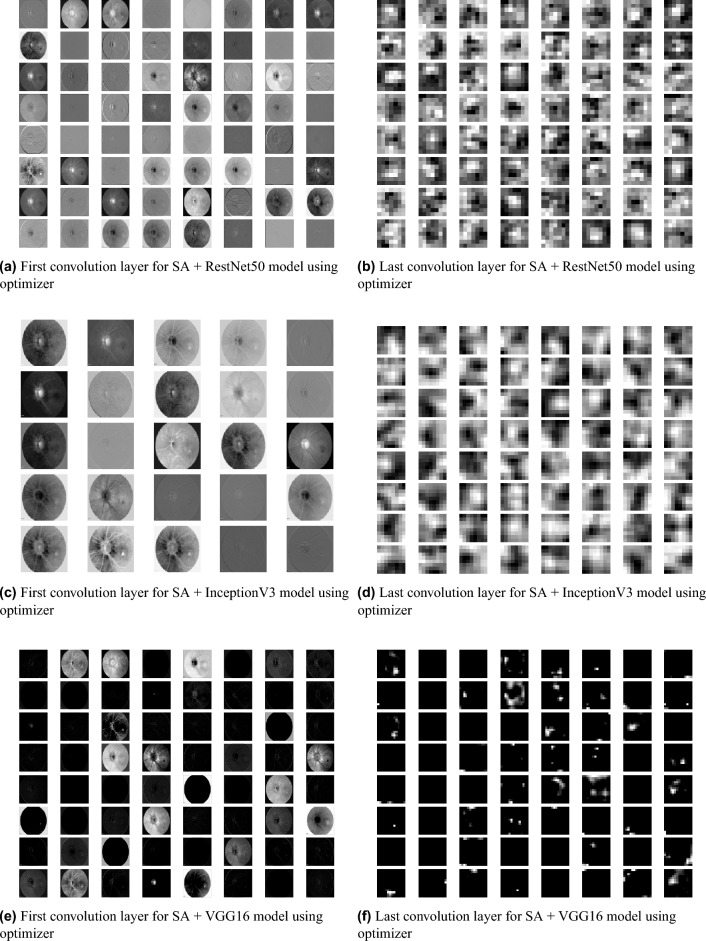
Feature Map visualization of first and last convolution layer of ResNet50, InceptionV3, and VGG16 pre-trained model after being integrated with SA model.

**Figure 5:**
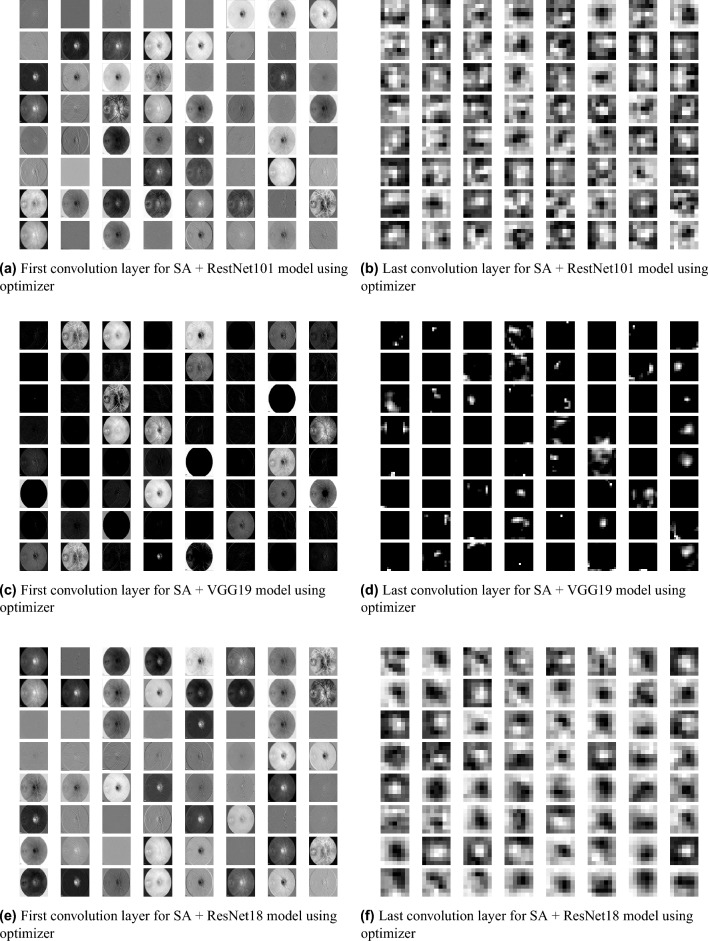
Feature Map visualization of first and last convolution layer of ResNet101, VGG19, and ResNet18 pre-trained model after being integrated with SA model.

**Figure 6:**
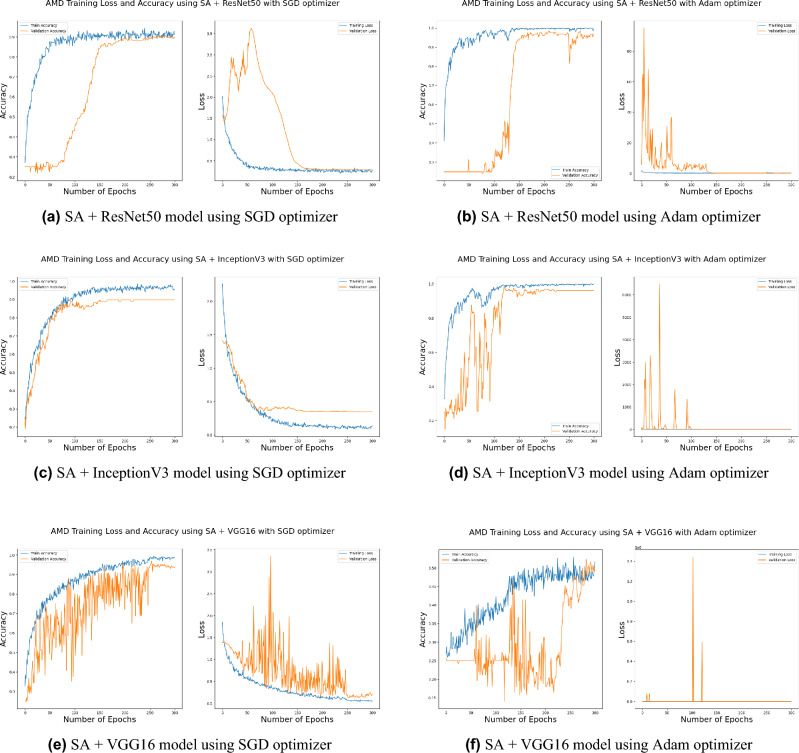
Plot diagrams of loss and accuracy records over 300 epochs for SA model integrated with InceptionV3, ResNet50, and VGG16 models using SGD and Adam optimizer.

**Figure 7:**
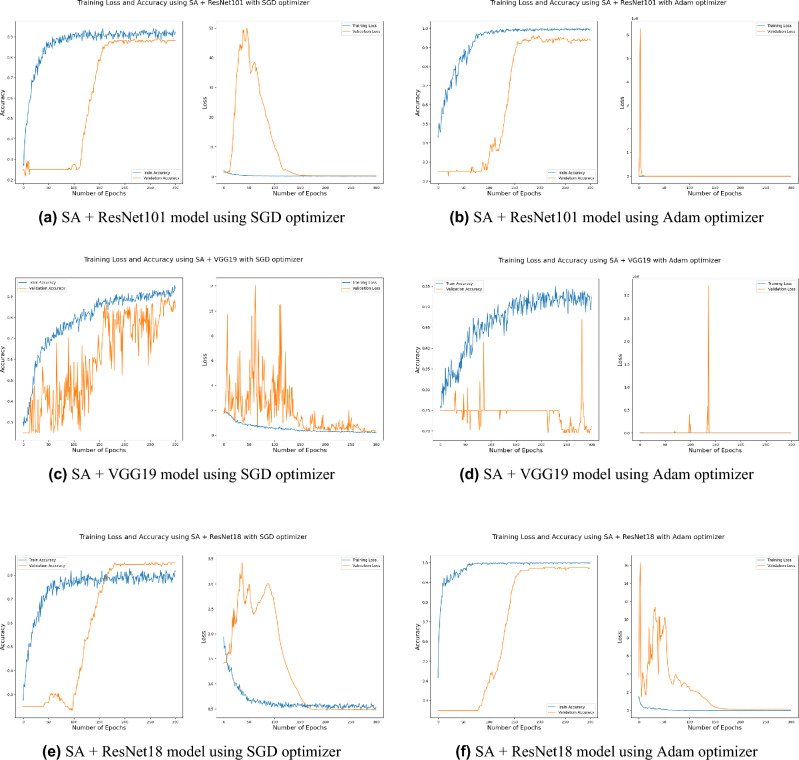
Plot diagrams of loss and accuracy records over 300 epochs for SA model integrated with ResNet101, VGG19, and ResNet18 models using SGD and Adam optimizer.

**Figure 8:**
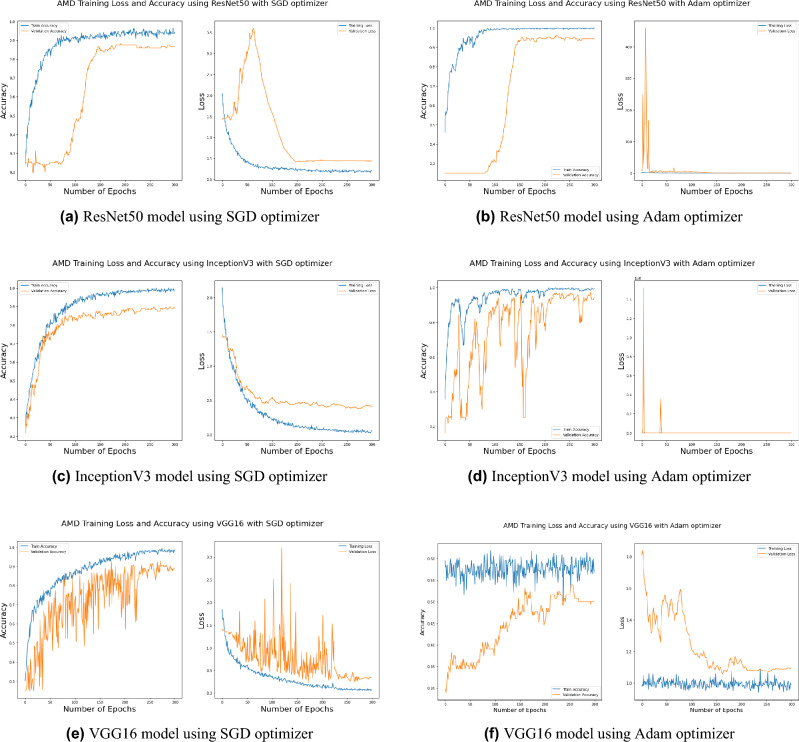
Plot diagrams of loss and accuracy records over 300 epochs for InceptionV3, ResNet50, and VGG16 standalone models using SGD and Adam optimizer.

**Figure 9:**
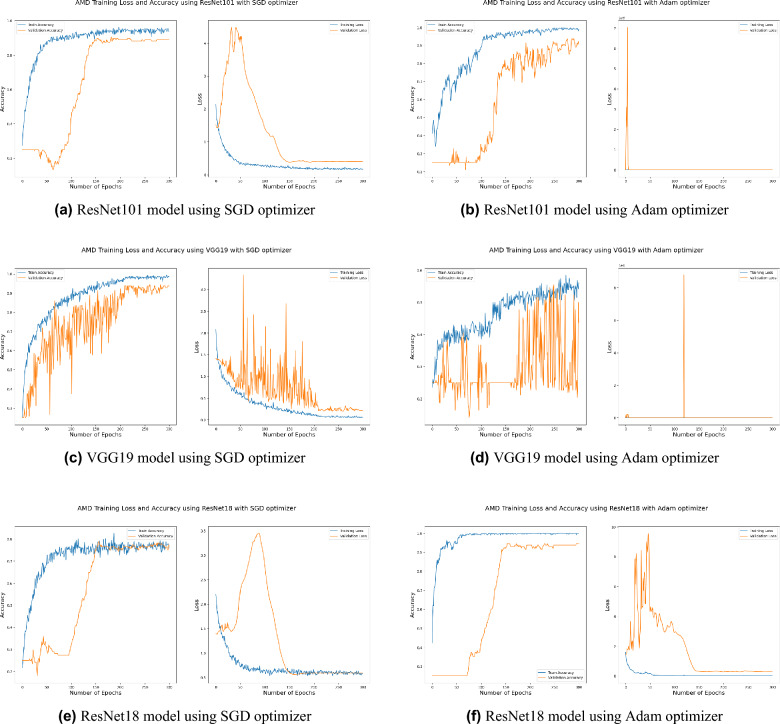
Plot diagrams of loss and accuracy records over 300 epochs for ResNet101, VGG19, and ResNet18 standalone models using SGD and Adam optimizer.

Distinguishing between the normal healthy retina and AMD different grades recorded the best performance when using our proposed integrated model compared to the other models. This is shown in Table 5 and Figs. 3, 6, 7, 8 and 9 plots the loss and accuracy recorded for the experimental models being integrated with SA and standalone respectively. Figures 6a,c,e and 7a,c,e shows the loss and accuracy for ResNet50, InceptionV3, VGG16, ResNet101, VGG19, and ResNet18 integrated with SA model using SGD optimizer respectively, while Figs. 6b,d,f, 7b and 9d,f shows the loss and accuracy for ResNet50, InceptionV3, VGG16, ResNet101, VGG19, and ResNet18 integrated with SA model using Adam optimizer respectively. Figures 8a,c,e and 9a,c,e shows the loss and accuracy for ResNet50, InceptionV3, VGG16, ResNet101, VGG19, and ResNet18 standalone pre-trained models using SGD optimizer respectively, while Figs. 8b,d,f and 9b,d,f shows the loss and accuracy for ResNet50, InceptionV3, VGG16 ResNet101, VGG19, and ResNet18 standalone pre-trained model using Adam optimizer respectively (Figs. 4, 5, 6).

SGD^48^ and Adam^49^ are significant optimization techniques used in machine learning for updating the weights of a neural network during training where the latter is considered as a hybrid combination of RMSProp and SGD with momentum^49^. SGD is a straightforward optimization approach that updates the neural network weights in the direction of the loss function’s negative gradient with respect to the weights. It randomly chooses a subset of the training data for every update, reducing the optimization’s computational cost. The choice of optimization algorithm depends on the problem being solved as well as the computing resources available. SGD is simple and computationally efficient, whereas Adam is more complex, but can achieve faster convergence on larger datasets and more complex studies^50^. According to the outcomes of applying the Bayesian optimization approach to detect the optimal hyperparameter tuning, the top nominated optimizers for tackling our problem were SGD and Adam optimizers with a batch size of 64 and 32 respectively as shown in Table 6 (Figs. 7, 8, 9).

**Figure 10:**
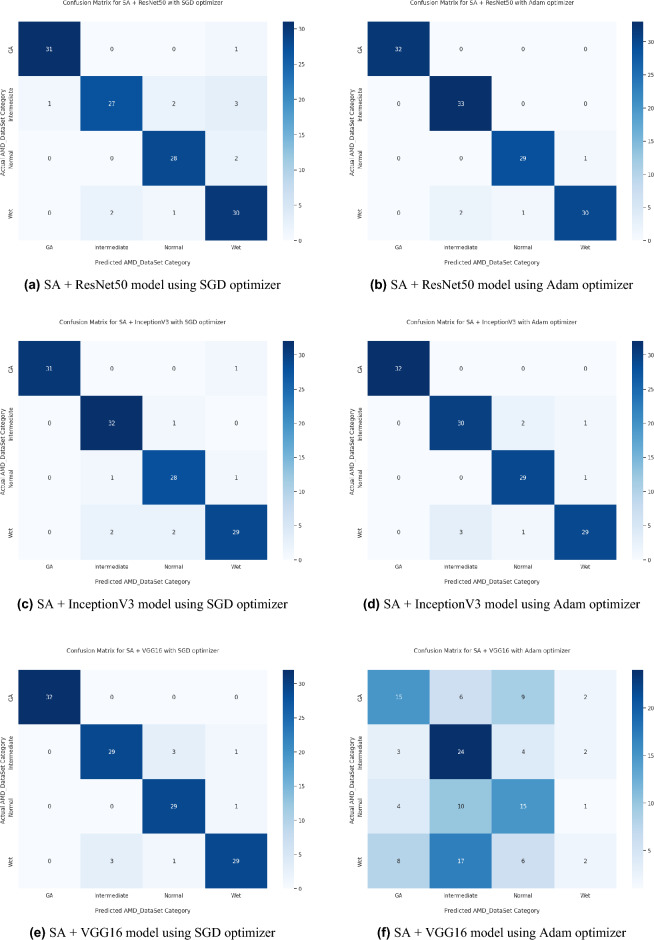
Confusion matrices of SA model integrated with InceptionV3 model, ResNet50 model, and VGG16 model using SGD and Adam optimizer.

**Figure 11:**
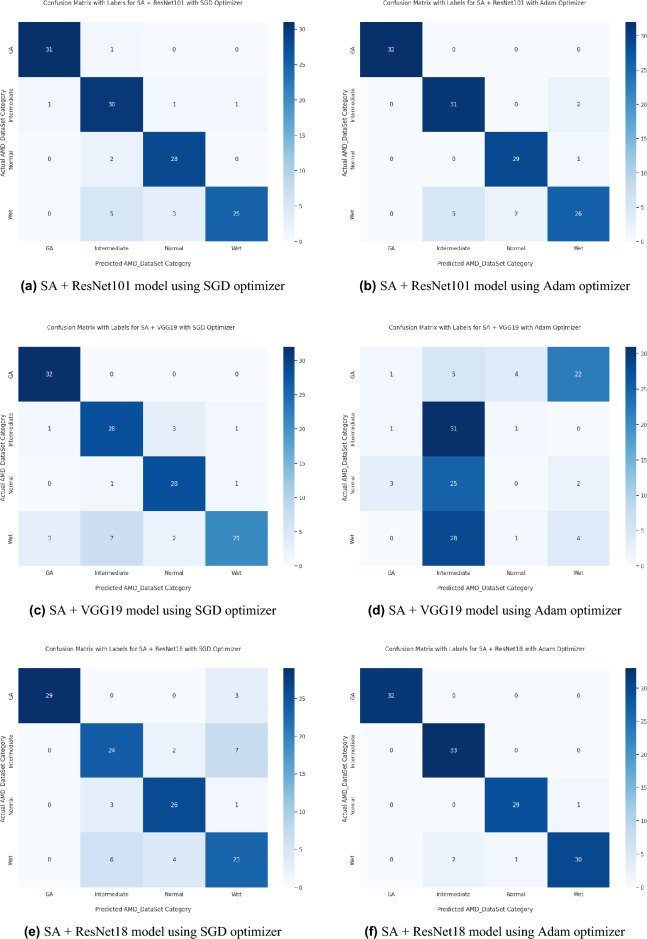
Confusion matrices of SA model integrated with ResNet101, VGG19, and ResNet18 models using SGD and Adam optimizer.

**Figure 12:**
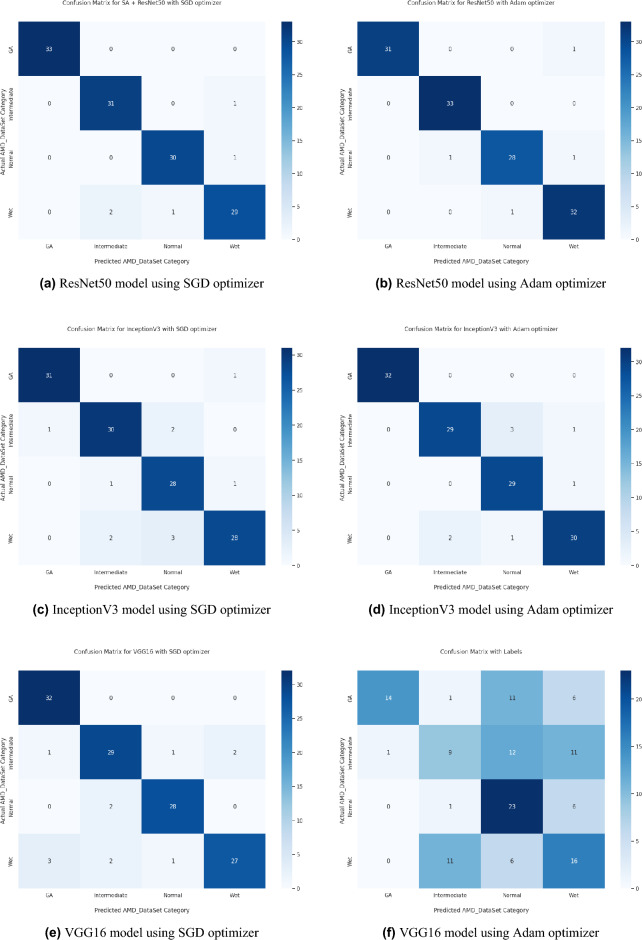
Confusion matrices of InceptionV3, ResNet50, and VGG16 standalone models using SGD and Adam optimizer.

**Figure 13:**
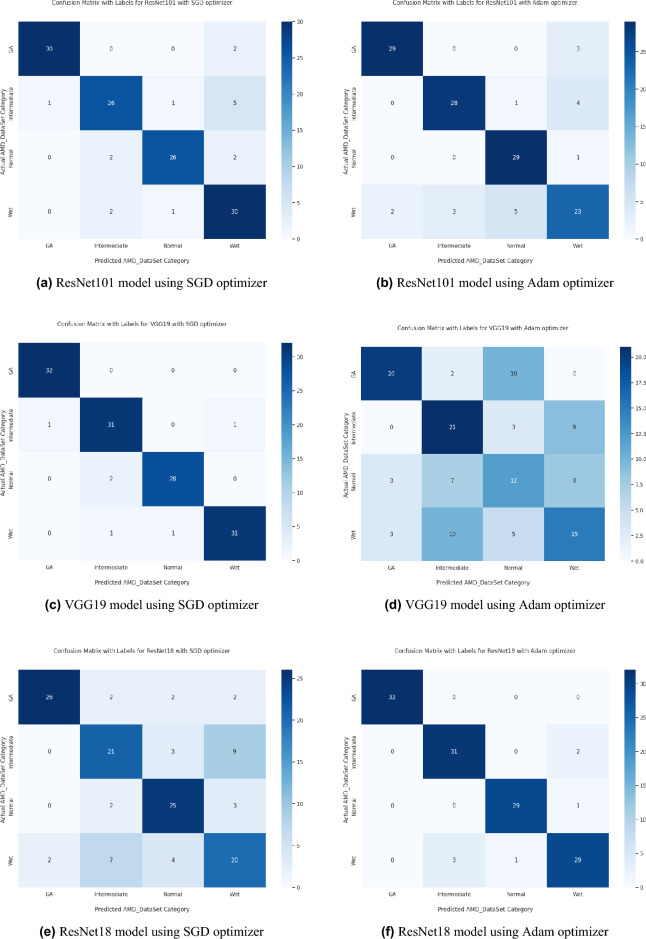
Confusion matrices of ResNet101, VGG19, and ResNet18 standalone models using SGD and Adam optimizer.

**Figure 14:**
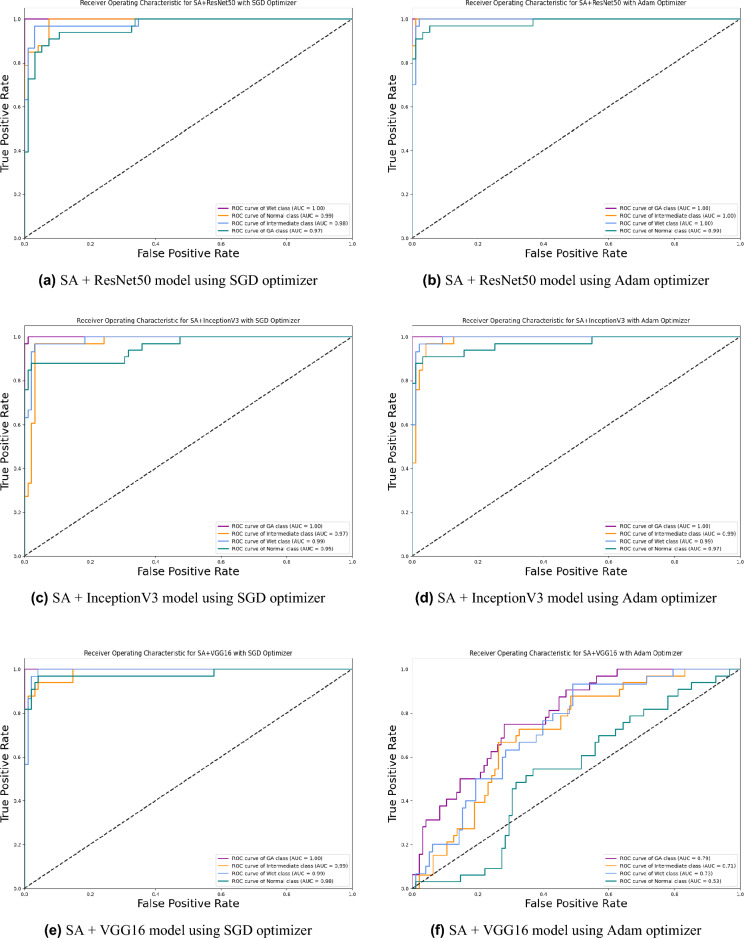
ROC of SA model integrated with InceptionV3, ResNet50, and VGG16 models using SGD and Adam optimizer.

**Figure 15:**
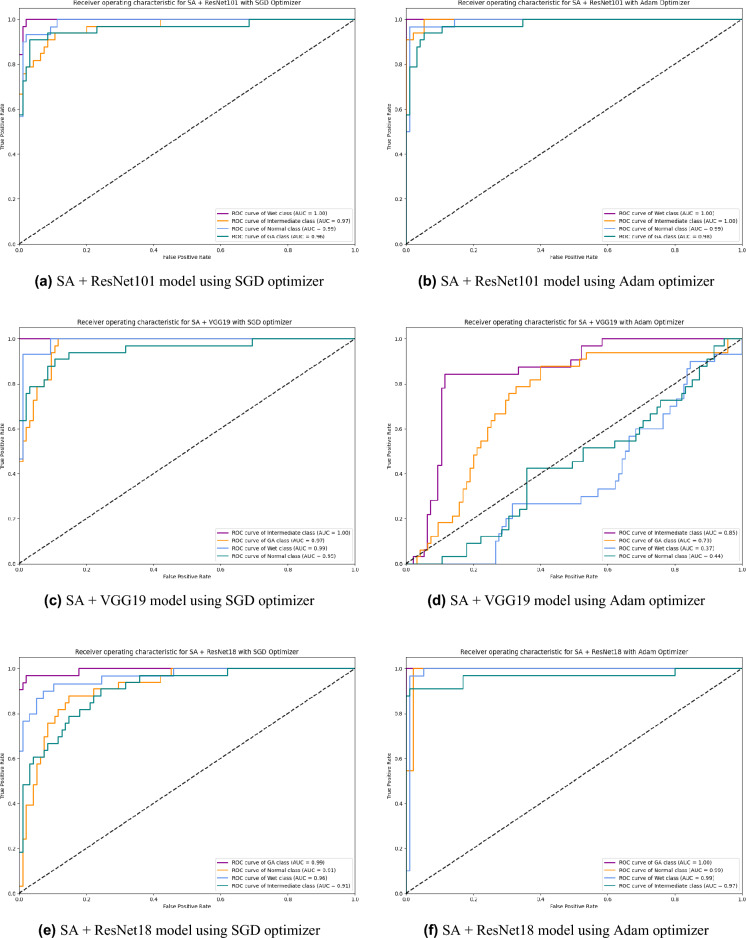
ROC of SA model integrated with ResNet101, VGG19, and ResNet18 models using SGD and Adam optimizer.

**Figure 16:**
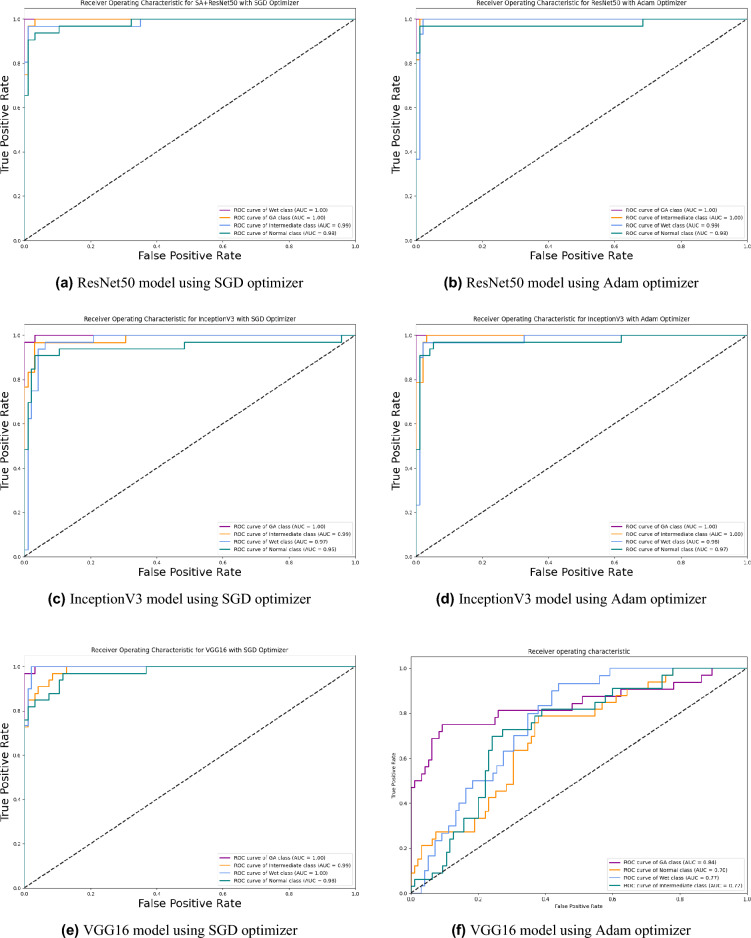
ROC of InceptionV3, ResNet50, and VGG16 standalone model using SGD and Adam optimizer.

**Figure 17:**
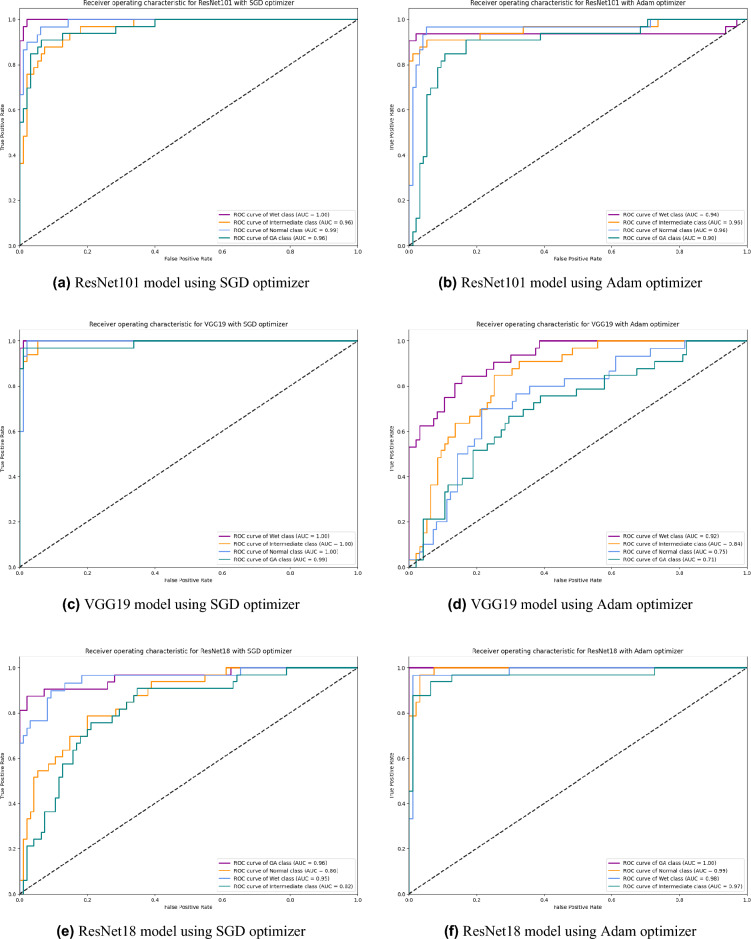
ROC of ResNet101, VGG19, and ResNet18 standalone models using SGD and Adam optimizer.

Based on our study, SGD proved to be a better optimization technique compared with Adam optimizer, the results are shown in Table 5 and Fig. 3. For every experiment, we started the learning rate value by 0.001 that adapted and reduced its value automatically, while to ensure fair experimental results we fixed any other hyper-parameter and set it to default except for batch size set to 64 over 300 epochs. Performance metrics of the trained models are shown in Tables 1 and 2 for SGD and Adam optimizers respectively, were computed based on the overall true-positives (TP), true-negatives (TN), false-positives (FP) and false-negatives (FN). The overall performance metrics and parameters are shown in Table 8 for using SGD optimizer and Table 9 for using the Adam optimizer. The confusion matrices is shown in Figs. 10 and 11 for the experimental models being integrated with SA, where Figs. 10a,c,e and 11a,c,e shows the confusion matrix for ResNet50, InceptionV3, VGG16, ResNet101, VGG19, and ResNet18 integrated with SA model using SGD optimizer respectively, while Figs. 10b,d,f and 13b,d,f shows the confusion matrix for ResNet50, InceptionV3, VGG16, ResNet101, VGG19, and ResNet18 integrated with SA model using Adam optimizer respectively. Figures 12 and 13 shows the confusion matrices for the standalone pre-trained models, where Figs. 12a,c,e and 13a,c,e shows the confusion matrix for ResNet50, InceptionV3, VGG16, ResNet101, VGG19, and ResNet18 standalone pre-trained models using SGD optimizer respectively, while Figs. 12b,d,f and 13b,d,f shows the confusion matrix for ResNet50, InceptionV3, VGG16, ResNet101, VGG19, and ResNet18 standalone pre-trained model using Adam optimizer respectively. The receiver operating characteristic (ROC) curves for all of the trained models are plotted in Figs. 14, 15, 16 and 17 for the experimental models being integrated with SA and standalone respectively. Figures 14a,c,e and 17a,c,e shows the ROC for ResNet50, InceptionV3, VGG16, ResNet101, VGG19, and ResNet18 integrated with SA model using SGD optimizer respectively, while Figs. 14b,d,f and 17b,d,f shows the ROC for ResNet50, InceptionV3, VGG16, ResNet101, VGG19, and ResNet18 integrated with SA model using Adam optimizer respectively. Figures 16a,c and 16e, 17a,c,e shows the ROC for ResNet50, InceptionV3, VGG16, ResNet101, VGG19, and ResNet18 standalone pre-trained models using SGD optimizer respectively, while Figs. 16b,d,f and 17b,d,f shows the ROC for ResNet50, InceptionV3, VGG16, ResNet101, VGG19, and ResNet18 standalone pre-trained model using Adam optimizer respectively. From the recorded results shown in Tables 1, 2, 8 and 9, it was clear that ResNet50 recorded the most promising performance metrics during training and testing phases by either using SGD or Adam optimizers concerning precision or positive predictive value (PPV), sensitivity or recall or true positive rate (TPR), and specificity or true negative rate (TNR) results. We applied 10-fold, 5-fold, and 3-fold cross-validation techniques for the pre-trained models integrated with SA using SGD optimizer or Adam optimizer to find the optimized performance as shown in Tables 3 and 4, comparing the results recorded for accuracy by training models in each k-fold. We also examined the proposed model with batch sizes 16, 32, and 128 as shown in Table 7 where it was observed that using the SGD optimizer recorded the highest accuracy value of 96.2% with batch size 64 although using the Adam optimizer with the same experimental environment recorded higher accuracy the cross-validation results promotes to using of SGD as shown in Table 3.

**Table 1:** Statistical analysis representation for precision, recall, and F1-score of Normal retina and different AMD grades for all of the training models using SGD optimizer.

	Normal	Intermediate	GA	Wet
SA + ResNet50				
Precision	0.97	0.94	1.00	0.94
Recall	0.97	0.97	1.00	0.91
F1-Score	0.97	0.95	1.00	0.92
ResNet50				
Precision	0.93	0.78	0.94	0.8
Recall	0.84	0.9	0.94	0.75
F1-Score	0.89	0.84	0.94	0.77
SA + VGG16				
Precision	0.93	0.89	0.79	0.97
Recall	0.93	0.94	1.00	0.88
F1-Score	0.93	0.91	0.98	0.92
VGG16				
Precision	0.96	0.88	0.89	0.88
Recall	0.9	0.88	0.97	0.85
F1-Score	0.93	0.88	0.93	0.86
SA + Inception				
Precision	0.91	0.89	1.00	0.97
Recall	0.97	0.97	0.97	0.85
F1-Score	0.97	0.93	0.98	0.9
Inception				
Precision	0.85	0.88	0.97	0.93
Recall	0.93	0.91	0.97	0.82
F1-Score	0.89	0.9	0.97	0.87
SA + ResNet101				
Precision	0.88	0.79	0.97	0.96
Recall	0.93	0.91	0.97	0.76
F1-Score	0.90	0.85	0.97	0.85
ResNet101				
Precision	0.93	0.87	0.97	0.77
Recall	0.87	0.79	0.94	0.91
F1-Score	0.90	0.83	0.95	0.83
SA + VGG19				
Precision	0.85	0.78	0.89	0.91
Recall	0.93	0.85	1.00	0.64
F1-Score	0.89	0.81	0.94	0.75
VGG19				
Precision	0.97	0.91	0.97	0.97
Recall	0.93	0.94	1.00	0.94
F1-Score	0.95	0.93	0.98	0.95
SA + ResNet18				
Precision	0.81	0.73	1.00	0.68
Recall	0.87	0.73	0.91	0.70
F1-Score	0.84	0.73	0.95	0.69
ResNet18				
Precision	0.74	0.66	0.93	0.59
Recall	0.83	0.64	0.81	0.61
F1-Score	0.78	0.65	0.87	0.60

**Table 2:** Statistical analysis representation for precision, recall, and F1-score of Normal retina and different AMD grades for all of the training models using Adam optimizer.

	Normal	Intermediate	GA	Wet
SA + ResNet50				
Precision	0.97	0.94	1.00	1.00
Recall	1.00	1.00	1.00	0.91
F1-Score	0.98	0.97	1.00	0.95
ResNet50				
Precision	0.97	0.97	1.00	0.89
Recall	0.93	0.97	0.94	0.97
F1-Score	0.95	0.97	0.97	0.93
SA + VGG16				
Precision	0.46	0.45	0.5	0.5
Recall	0.6	0.76	0.47	0.06
F1-Score	0.52	0.57	0.48	0.11
VGG16				
Precision	0.44	0.41	0.93	0.41
Recall	0.77	0.27	0.44	0.48
F1-Score	0.56	0.33	0.6	0.44
SA + Inception				
Precision	0.94	0.91	1.00	0.94
Recall	0.97	0.94	1.00	0.88
F1-Score	0.95	0.93	1.00	0.91
Inception				
Precision	0.94	0.94	1.00	0.97
Recall	0.97	0.97	1.00	0.91
F1-Score	0.95	0.96	1.00	0.94
SA + ResNet101				
Precision	0.94	0.86	1.00	0.90
Recall	0.97	0.94	1.00	0.79
F1-Score	0.95	0.90	1.00	0.84
ResNet101				
Precision	0.83	0.90	0.94	0.74
Recall	0.97	0.85	0.91	0.70
F1-Score	0.89	0.88	0.92	0.72
SA + VGG19				
Precision	0.00	0.35	0.20	0.14
Recall	0.00	0.94	0.03	0.12
F1-Score	0.00	0.51	0.05	0.13
VGG19				
Precision	0.40	0.53	0.77	0.47
Recall	0.40	0.64	0.62	0.45
F1-Score	0.40	0.58	0.69	0.46
SA + ResNet18				
Precision	0.97	0.94	1.00	0.97
Recall	0.97	1.00	1.00	0.91
F1-Score	0.97	0.97	1.00	0.94
ResNet18				
Precision	0.97	0.91	1.00	0.91
Recall	0.97	0.94	1.00	0.88
F1-Score	0.97	0.93	1.00	0.89

**Table 3:** Summary of K-fold cross-validation over the experimental models (ResNet50, InceptionV3, and VGG16) shows accuracy mean and standard deviation recorded among 10-folds, 5-folds, and 3-folds cross-validation for every trained model using SGD and Adam optimizers.

	SA + ResNet50		SA + VGG16		SA + InceptionV3	
	Mean	STD	Mean	STD	Mean	STD
10-Folds						
Adam	99.8%	+/- 0.38%	47.93%	+/- 26.46%	27.24%	+/- 10.59%
SGD	96.55%	+/- 1.26%	59.54%	+/- 37.00%	96.44%	+/- 1.20%
5-Folds						
Adam	88.97%	+/- 1.17%	71.72%	+/- 9.35%	28.89%	+/-8.60%
SGD	96.55%	+/- 1.03%	97.01%	+/- 1.56%	96.09%	+/- 0.56%
3-Folds						
Adam	87.36%	+/- 1.63%	73.56%	+/- 5.71%	61.30%	+/- 21.53%
SGD	95.02%	+/- 1.05%	73.56%	+/- 7.68%	95.79%	+/- 1.43%

**Table 4:** Summary of K-fold cross-validation over the experimental models (ResNet101, VGG19, and ResNet18) shows accuracy mean and standard deviation recorded among 10-folds, 5-folds, and 3-folds cross-validation for every trained model using SGD and Adam optimizers.

	SA + ResNet101		SA + VGG19		SA + ResNet18	
	Mean	STD	Mean	STD	Mean	STD
10-Folds						
Adam	19.94%	+/- 7.59%	52.04%	+/- 26.94%	27.54%	+/- 9.59%
SGD	34.96%	+/- 24.08%	22.00%	+/- 7.67%	88.43%	+/- 6.29%
5-Folds						
Adam	24.59%	+/- 5.18%	71.81%	+/- 13.91%	22.67%	+/- 6.06%
SGD	24.98%	+/- 7.98%	30.24%	+/- 10.17%	86.62%	+/- 2.42%
3-Folds						
Adam	66.02%	+/- 29.23%	67.18%	+/- 1.97%	77.22%	+/- 27.65%
SGD	50.32%	+/- 29.67%	43.50%	+/- 26.16%	81.85%	+/- 5.50%

### Explainable retina maps

**Figure 18:**
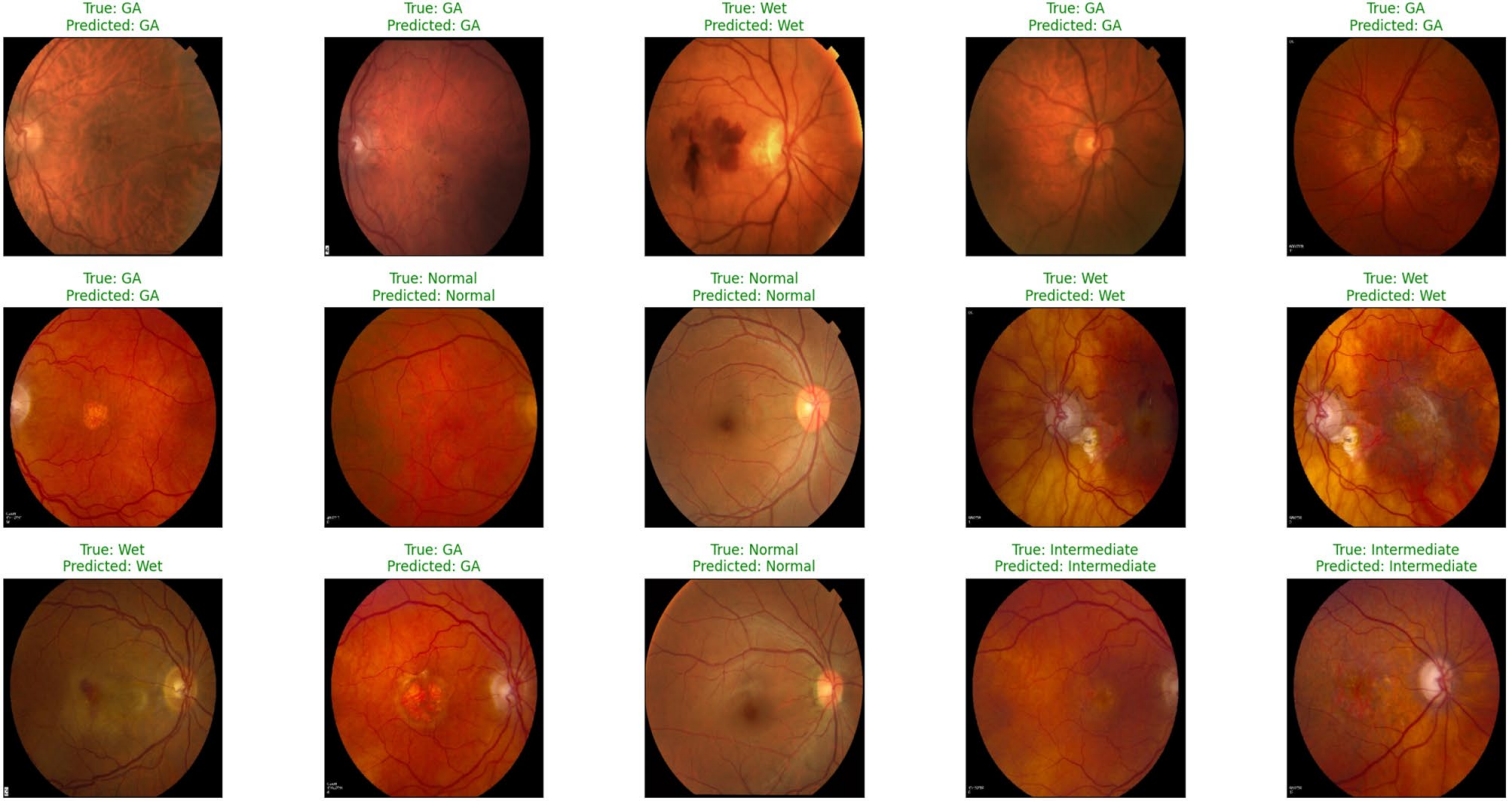
Sample prediction outputs of SA + ResNet50 model using SGD optimizer successfully detected AMD different grads.

We used a feature map to ensure the availability of information and visualize feature propagation among convolution layers till the last layer. Figure 4 shows feature maps visualization of the first and last convolution layer of the proposed model and SA integrated with other pre-trained models, where Fig. 4a shows the output of its 64 filters first convolution layer of ResNet50 pre-trained model integrated with SA while its last convolution layer shown in Fig. 4b displays the output of 64 filters. Similarly, for SA integrated with InceptionV3 pre-trained model, we displayed its 25 filters of first convolution layers as shown in Fig. 4c while its output is shown in Fig. 4d where we display the output of 64 kernels out of 192 filters. For the VGG16 pre-trained model being integrated with SA, Fig. 4e,f show the output of the top 64 filters for the first and last convolution layers, respectively. Figure 5a,c,e show the output of the top 64 filters for the first convolution layer of ResNet101, VGG19, and ResNet18 pre-trained models being integrated with SA respectively, while Fig. 5b,d,f show the output of top 64 filters for the last convolution layer of ResNet101, VGG19, and ResNet18 pre-trained models being integrated with SA respectively. The predicted output using the proposed model is shown in Fig. 18, where it successfully discriminates between AMD different grading.

## Discussion

In this study, we propose an integrated deep learning model capable of recognizing and differentiating between the normal retina and various clinical grades of AMD (intermediate, GA, or Wet AMD) successfully with high accuracy using retinal fundus images. We faced limitations to optimize the performance and build high accuracy model because of a limited number and variety of fundus dataset image samples; we applied transfer learning approach and compared the results between training standalone ResNet50, VGG16, InceptionV3, ResNet101, VGG19, ResNet18 pre-trained models and integrating each of these models with SA model, where SA is the model trained for accepting fundus images of different sizes and dimensions and producing scaled output image of $224 \times 224$ px size. Many public datasets contain medical fundus images covering various retinal diseases such as AMD, diabetic retinopathy, glaucoma, and cataracts. Most of the datasets for AMD such as iChallenge-AMD^51^, ODIR-2019^52^, Automated Retinal Image Analysis ARIA^53^, and STARE^54^ classify images into AMD and normal retina. Hence, it was hard to use any of these datasets in either training, testing, or evaluating the proposed model (Tables 5, 6, 7).

**Table 5:** Accuracy values for ResNet50, InceptionV3, VGG16, ResNet101, VGG19, ResNet18 standalone models, and integrated with SA model using SGD and Adam optimizers.

	SGD (%)	Adam (%)
SA + ResNet50	96.20	97.70
ResNet50	91.70	95.50
SA +VGG16	93.90	47
VGG16	90.90	48.5
SA + InceptionV3	93.90	94.70
InceptionV3	90.90	96.20
SA + ResNet101	89.4	92.4
ResNet101	87.9	85.6
SA +VGG19	85.6	27.3
VGG19	95.5	53
SA +ResNet18	80.3	96.7
ResNet18	72.2	94.7

**Table 6:** The outcomes of the Bayesian optimization approach indicate the optimal hyperparameters tuning in terms of the optimizer and batch size to achieve the best performance.

Optimizer	Batch size
SGD	64
SGD	32
adam	32
rmsprop	16
SGD	16
nadam	16
adadelta	32

**Table 7:** Accuracy values of ResNet50 integrated with SA model associated with different batch sizes 16, 32, 64, and 128 using SGD and Adam optimizers.

Batch size	SGD (%)	Adam (%)
16	95.2	82
32	94.5	92
**64**	**96.2**	**97.7**
128	25.5	26

Significant values are in [bold].

Despite these limitations, our model classified the AMD grades successfully and recorded an accuracy of 96.2% for integrating the SA model with the ResNet50 model using SGD optimizer although using Adam optimizer recorded an accuracy of 97.7%. The best model was determined based on the results from Tables 1, 2, 8 and 9 and applying several deep learning methodologies such as k-fold cross validation recorded in Table 3 to ensure high model performance and by evaluating the model using 3-folds, 5-folds and 10-folds to determine optimal performance and decide the best model. By applying data augmentation, the dataset was sufficient to demonstrate the feasibility of our proposed deep learning model to distinguish AMD grades using fundus images. We examined the integrated model and tried different optimization like Adam and SGD which proved to be the best optimization technique in our case study.

**Table 8:** Quantitative comparison between the proposed model (SA + ResNet50) and other classification networks (InceptionV3, VGG16, ResNet101, VGG19, and ResNet18 each with or without SA, and also ResNet50 without SA). For all of them we use SGD Optimizer during the training phase.

	TP	TN	FP	FN	Precision	Sensitivity or Recall (%)	F1-Score (%)	Specificity (%)	Accuracy (%)	AUC (%)
SA + ResNet50	127	392	4	5	0.9695	96.21	96.6	99	96.2	98.83
ResNet50	118	386	10	14	0.92	89.4	90.8	96.6	91.7	98.3
SA +VGG16	124	388	8	8	0.939	93.9	93.9	98	93.9	98.87
VGG16	118	385	11	14	0.9147	89.4	90.4	97.2	90.9	98.8
SA + InceptionV3	123	388	8	9	0.93	93.2	93.5	98	93.9	98
InceptionV3	120	384	12	12	0.909	90.9	90.9	97	90.9	97
SA + ResNet101	114	385	11	18	0.912	86.36	97.2	88.7	89.4	97.86
ResNet101	115	384	12	17	0.9055	87.12	97	88	87.9	98.09
SA +VGG19	112	380	16	20	0.875	84.85	96	86.2	85.6	97.6
VGG19	125	391	5	7	0.9615	94.7	98.7	95.4	95.5	99.22
SA + ResNet18	100	378	18	32	0.8475	75.76	80	95.5	80.3	95.32
ResNet18	89	370	26	43	0.7739	67.42	72.1	93.4	72.7	91.41

**Table 9:** Quantitative comparison between (SA + ResNet50) model and other classification networks (InceptionV3, VGG16, ResNet101, VGG19, and ResNet18 each with or without SA, and also ResNet50 without SA). For all of them we use Adam Optimizer during the training phase.

	TP	TN	FP	FN	Precision	Sensitivity or Recall (%)	F1-Score (%)	Specificity (%)	Accuracy (%)	AUC (%)
SA + ResNet50	129	393	3	3	0.977	97.7	97.7	99.2	97.7	99
ResNet50	126	390	6	6	0.9545	95.5	95.8	98.5	95.5	98.7
SA +VGG16	26	368	28	106	0.4815	19.7	28	94.5	47	71
VGG16	52	357	39	80	0.57	39.4	46.6	90.2	48.5	77.65
SA + InceptionV3	125	389	7	7	0.947	94.7	94.7	98.2	94.7	98
InceptionV3	126	391	5	6	0.96	95.5	95.8	98.7	96.2	98.76
SA + ResNet101	122	387	9	10	0.9313	92.42	97.7	92.8	92.4	98.62
ResNet101	113	377	19	19	0.8561	85.61	95.2	85.6	85.6	94.67
SA +VGG19	24	363	33	108	0.4211	18.18	91.7	25.4	27.3	51.86
VGG19	40	377	19	92	0.678	30.30	95.2	41.9	53	80.39
SA + ResNet18	128	392	4	4	0.9697	96.97	97	99	96.7	98.41
ResNet18	125	389	7	7	0.947	94.7	94.7	98.2	94.7	98.63

The pre-trained model represented in ResNet50 proved to be more efficient either integrated with the SA model or standalone whether using SGD or Adam optimizer. It recorded the best-fit model to our study according to cross-validation technique results recorded in Table 3. During the training phase it recorded accuracy that is comparatively 3% accuracy higher than using VGG16 and InceptionV3 models when being integrated with SA model. Compared with ResNet101, VGG19, and ResNet18; the proposed model recorded higher accuracy by more than 6%, 10%, and 15% respectively. It recorded 91.7% accuracy when trained as a standalone model. Although VGG16 pre-trained model recorded performance metrics like InceptionV3 pre-trained model using SGD, and VGG19 pre-trained model recorded acceptable results using SGD both VGG16 and VGG19 recorded the lowest results using Adam optimizer either as a standalone model or integrated with the SA model. InceptionV3 recorded good performance metrics during the training phase. However, it was excluded due to cross-validation technique results similar, to ResNet101 and ResNet18.

## Conclusion and future work

In this study, we have proposed an integrated model for scaling input images and distinguishing between normal retinas and AMD grades using color fundus images. Our approach involves two stages. The first stage is a custom auto-encoder-based model that aims to resize the input images to $224 \times 224 \times 3$ dimensions, then considers any needed data preprocessing, and then feeds its output to the second stage that aims to classify its input into normal retinas, intermediate AMD, GA and wet AMD grades using ResNet50 pre-trained model. The proposed model is trained on the color fundus images dataset provided by the CATT Study Group. We compared our proposed model performance against different pre-trained models either standalone or integrated with our SA model. We validate our approach using a cross-validation technique that proves our proposed model is the best model performance.

For future work, we plan to integrate the scale adapting network with other systems that diagnose other retinal disease, such as diabetic retinopathy, and with other networks that work on different imaging modalities. Also, we plan to expand the study by collecting data from additional cohorts that include subjects from a wider range of institutions and geographic areas globally.

## Data Availability

The datasets used and analysed during the current study will be available from the corresponding author on reasonable request.
